# Sequential Development of Chronic Lymphocytic Leukemia (CLL) in a Chronic Myeloid Leukemia (CML) Patient Treated With Nilotinib: A Case Report

**DOI:** 10.7759/cureus.91223

**Published:** 2025-08-29

**Authors:** Ahmed Elazab, Maged Saafan, Noreen Elbayoumi

**Affiliations:** 1 Hematology, Oncology Center, Mansoura University, Mansoura, EGY

**Keywords:** case report, chronic lymphocytic leukemia, chronic myeloid leukemia, nilotinib, tyrosine kinase inhibitors (tki)

## Abstract

Chronic myeloid leukemia (CML) and chronic lymphocytic leukemia (CLL) are distinct clonal hematologic malignancies that rarely occur in the same patient. We report the case of a 52-year-old male with CML who achieved a sustained major molecular response on the tyrosine kinase inhibitor (TKI) nilotinib and who subsequently developed asymptomatic CLL during follow-up. The CML was well-controlled (BCR-ABL1 < 0.1%), and leukocytosis was investigated and found to be due to a monoclonal B-cell population consistent with CLL. This unusual sequential presentation highlights the importance of evaluating unexplained leukocytosis even in a patient with well-managed CML. We discuss potential pathophysiologic mechanisms, including whether TKI therapy could influence clonal evolution and review relevant literature. The patient continues nilotinib therapy with ongoing CML remission and remains under observation for CLL, underscoring a conservative management approach given the indolent nature of his CLL. This case adds to the sparse literature on CML and CLL co-occurrence and raises awareness of secondary hematologic malignancies in TKI-treated CML patients.

## Introduction

The coexistence of CML and CLL in the same individual is extremely rare, with only sporadic cases reported in the literature [1-3]. CML is a myeloproliferative neoplasm driven by the BCR::ABL1 (breakpoint cluster region-AbLeson fusion gene) fusion tyrosine kinase, whereas CLL is a lymphoproliferative disorder of monoclonal CD5+ve B lymphocytes. Given their differing cell lineages and molecular drivers, CML and CLL are considered separate clonal diseases, and all documented cases of dual CML/CLL have shown the malignancies to arise from independent clones with different genomic profiles [1,4]. As such, the sequential occurrence of one after the other in a single patient is an exceptional clinical phenomenon.

Several classifications of CML/CLL co-occurrence have been described, including CML preceding CLL (as in our case) and CLL preceding CML. The pathophysiologic mechanisms underlying this association remain unclear [5]. One hypothesis is that the occurrence of a second leukemia is a stochastic event, given that both diseases typically affect older adults and may simply coincide by chance in rare instances [1]. Alternatively, there may be predispositional factors; for example, a germline genetic susceptibility or an altered bone marrow microenvironment that permits the emergence of a second malignant clone. It has also been suggested that CML or its treatment could create an immunologic alteration favoring a secondary lymphoproliferative disorder. TKIs like imatinib, and by extension second-generation TKIs such as nilotinib can interfere with T-lymphocyte function and hematopoietic progenitor differentiation, potentially impairing immune surveillance and encouraging the development of secondary hematologic malignancies [6]. Indeed, an analysis from the German CML Study IV noted a higher-than-expected incidence of non-Hodgkin lymphoma in patients with CML on TKI therapy [6], though overall data on secondary cancers in TKI-treated CML are mixed. Regardless of the cause, recognizing a secondary leukemia in CML patients is critical for proper management.

In this report, we present a case of sequential development of CLL in a patient with CML who was maintained on nilotinib. We describe the clinical course, diagnostic workup, and management and then discuss the implications of this case in the context of the existing literature. This case aims to alert clinicians to the potential for dual hematologic malignancies and to explore whether nilotinib or clonal interactions might have contributed to this unusual sequence.

## Case presentation

A 52-year-old man presented with a one-month history of persistent pain in the left hypochondrium. He denied fevers, night sweats, or weight loss. On examination, he was found to have splenomegaly, with the spleen palpable about four finger-breadths below the left costal margin. No significant lymphadenopathy was noted at presentation. Laboratory evaluation revealed a markedly elevated white blood cell count: the total leukocyte count was 210 × 10^9^/L, absolute lymphocyte count was 8 × 10^9^/L, hemoglobin was 10.2 g/dL, and platelet count was 239 × 10^9^/L. Peripheral blood smear review demonstrated features consistent with CML in the chronic phase, including left-shifted granulocytosis with circulating myelocytes and promyelocytes. Bone marrow aspirate and cytogenetic analysis confirmed the presence of the Philadelphia chromosome. Quantitative polymerase chain reaction (PCR) for BCR-ABL1 transcripts was positive, with a BCR::ABL1 transcript level of 73% international scale (IS), confirming the diagnosis of CML in chronic phase. The Sokal risk score was calculated as intermediate based on the patient’s counts and spleen size.

The patient was started on frontline therapy with nilotinib (300 mg twice daily), a second-generation TKI. After six months of therapy, repeat quantitative PCR showed a major molecular response (MMR), with BCR::ABL1 transcript level falling to 0.035% IS (indicating >3-log reduction from baseline). The patient’s blood counts normalized, and the spleen regressed in size. As our center is not adopting treatment-free remission (TFR) drug discontinuation, he continued nilotinib with regular monitoring. At approximately three years into therapy, during which he maintained consistent molecular response with BCR::ABL1 < 0.1%, a gradual leukocytosis was noted. His white cell count, which had been within normal range during molecular remission, increased to 20 × 10^9^/L. Importantly, concurrent BCR::ABL1 PCR at that time confirmed ongoing deep molecular remission of CML indicating that the rising leukocyte count was not due to CML recurrence.

**Figure 1:**
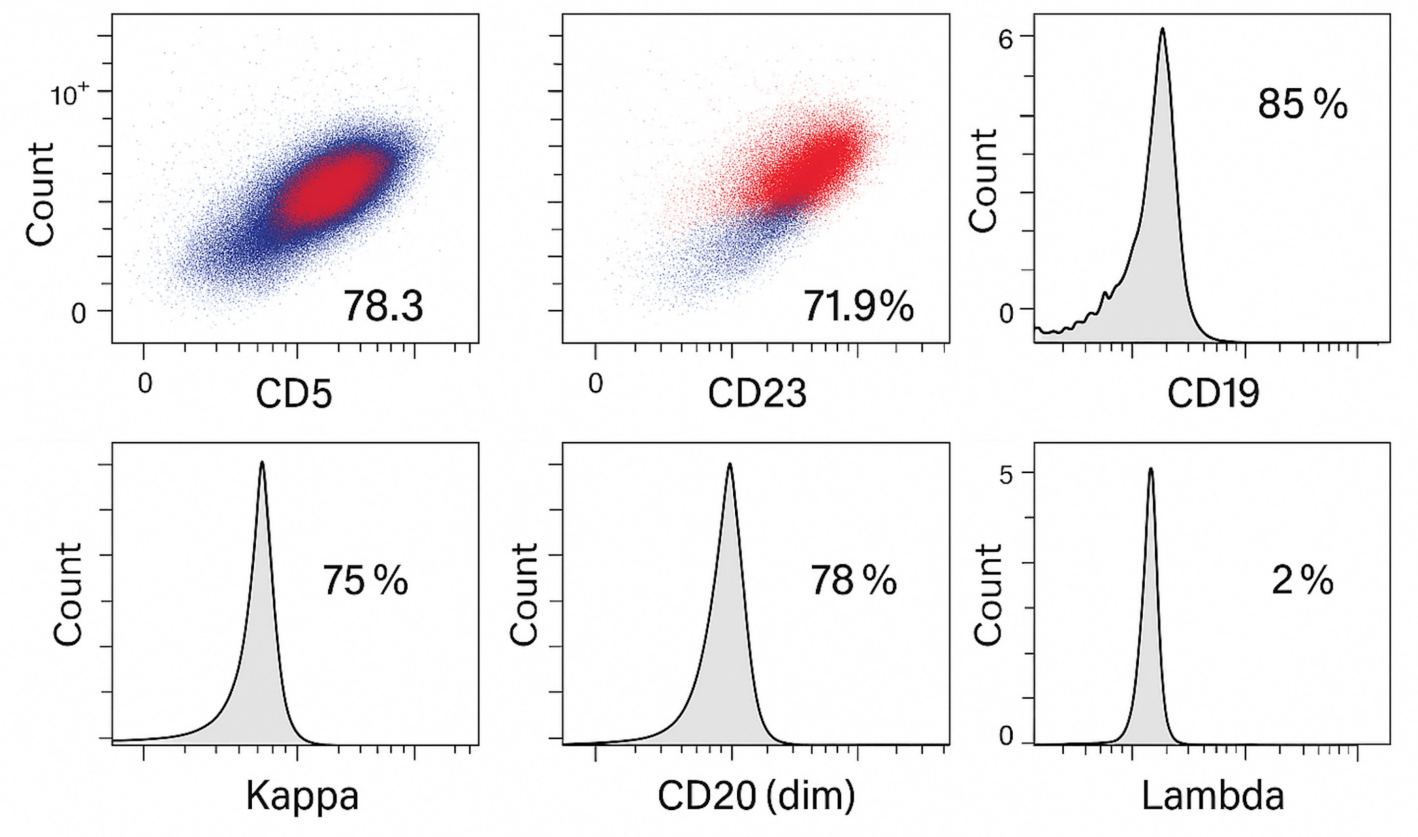
Flow cytometric immunophenotyping of peripheral blood leukocytes revealing a monoclonal B-cell population co-expressing CD5, CD19, CD20 (dim), and CD23, with restriction to kappa light chain, consistent with CLL immunophenotype Events were gated on CD19⁺ B cells, which comprised 85% of lymphocytes. The cells co-expressed CD5 (78.3%) and CD23 (71.9%), demonstrated dim CD20 expression (78%), and showed kappa light chain restriction (75%) with absent lambda expression (2%).

Given the unexplained leukocytosis in a patient with otherwise controlled CML, further evaluation was undertaken. Differential count showed an absolute lymphocytosis. Morphologically, the peripheral blood now demonstrated a predominant population of small mature-appearing lymphocytes. Flow cytometric immunophenotyping of peripheral blood leukocytes was performed, which revealed a monoclonal B-cell population co-expressing CD5, CD19, CD20 (dim), and CD23, with restriction to kappa light chain, consistent with CLL immunophenotype (Figure 1). Fluorescence in situ hybridization (FISH) analysis showed no deletion of the TP53 locus on 17p, and there was no evidence of del(11q) or trisomy 12. Immunoglobulin heavy chain mutation status and other molecular CLL risk markers were not assessed at that time due to nonavailability. Staging evaluations, including computed tomography of the neck, chest, abdomen, and pelvis, showed no bulky lymphadenopathy or organomegaly aside from the spleen, which was mildly enlarged at 13 cm, stable from prior imaging. The patient remained asymptomatic, and his hemoglobin level and platelet count were within normal ranges.

Overall, the findings were consistent with an asymptomatic early-stage CLL (Stage 0) on Rai classification in a patient with concurrently well-controlled CML. Given the lack of CLL-related symptoms or significant cytopenias, the patient did not meet the indications for immediate CLL treatment. He was thus managed with observation (“watch and wait”) for CLL while continuing nilotinib therapy to maintain his CML remission as per local policy. At the time of this report, approximately four years from the CML diagnosis, the patient remains in MMR on nilotinib and has experienced no progression of CLL - his lymphocyte count has fluctuated but without exponential growth, and he continues to be Rai stage 0 (low risk) for CLL. Periodic follow-up with blood counts, BCR::ABL1 PCR, and clinical examination is ongoing.

## Discussion

The sequential development of CLL in a patient with CML raises intriguing questions about pathogenesis and the potential interplay of two distinct hematologic malignancies. Only a few dozen cases of CML coexisting with CLL have been documented in the literature to date. In virtually all reported instances, the two leukemias represent separate clones rather than a transformation of one disease into the other. This is supported by cytogenetic and molecular findings: for example, CLL cells in such cases typically lack the Philadelphia chromosome or BCR::ABL1 fusion seen in CML, and conversely, the myelogenous cells do not carry CLL-specific immunophenotypic markers or immunoglobulin gene rearrangements. Thus, the scenario in our patient is best explained by two independent clonal neoplasms arising sequentially rather than one disease transformed into another.

The rarity of this co-occurrence naturally prompts speculation about whether it is simply coincidental or if there is a causal relationship. One consideration is the impact of long-term TKI therapy on hematopoiesis and immune surveillance. Imatinib, the prototypical BCR::ABL1 inhibitor, is known to have immunomodulatory effects, including inhibition of T-cell function and cytokine signaling pathways [7]. Nilotinib, as a second-generation TKI, is more selective for BCR::ABL1, but it shares some off-target kinase inhibition (e.g., discoidin domain receptor (DDR), KIT, and platelet-derived growth factor receptor alpha (PDGFR)) and could conceivably also affect immune cell homeostasis [8]. It has been suggested that TKIs might create a more permissive environment for a preexisting aberrant B-cell clone to expand. In our case, one could hypothesize that an indolent CLL clone was present at a very low level and not noticed until the CML was well-controlled. Once the dominant myeloid clone was suppressed by nilotinib and the marrow microenvironment shifted, the previously silent CLL clone may have gained a growth advantage and become detectable. This “unmasking” theory aligns with other reports in which CLL was diagnosed during sustained remission of CML.

Another angle to consider is whether CML and its treatment predispose patients to secondary malignancies in general. Population studies of CML patients on TKI therapy have yielded conflicting results. While some analyses show no significant increase in overall secondary cancer risk compared to the general population, there have been hints of increased incidence of certain malignancies, particularly lymphoid cancers [9]. The patient did not receive cytotoxic chemotherapy or radiation - his only cancer-directed therapy was nilotinib. Nilotinib’s known adverse effects include metabolic syndrome and vascular events, but it has not been strongly linked to therapy-related secondary neoplasms in clinical trials. Thus, any causal link between nilotinib and CLL remains speculative.

From a clinical management standpoint, the coexistence of CML and CLL can pose challenges, especially if both diseases are active and require treatment. Fortunately for our patient, his CLL has so far remained asymptomatic and at low tumor burden; as a result, there was no need for immediate therapy. Standard frontline treatment for CLL (if indicated) often involves either Bruton's tyrosine kinase inhibitors (e.g., ibrutinib, acalabrutinib) or BCL-2 inhibitors (venetoclax) ± anti-CD20 antibodies, depending on risk factors [9]. There is limited precedent for managing both CML and CLL concurrently, but a few case reports have described successful dual therapy. For example, one patient with CML on imatinib who developed high-risk CLL was treated concomitantly with ibrutinib; the combination was tolerated without significant additive toxicity [10]. Drug-drug interactions must be considered in such scenarios (imatinib and ibrutinib both share CYP3A4 metabolism, potentially affecting levels). In our case, since CLL therapy was not yet indicated, we continued nilotinib alone and monitored closely. If the CLL were to progress and require treatment, the optimal approach is not well-defined given the rarity of concurrent CML and CLL, and management must be individualized.

Our case emphasizes the importance of not attributing every laboratory change to the preexisting diagnosis. The discovery of a rising lymphocyte count in a CML patient in deep remission should prompt an evaluation for a second pathology rather than an automatic assumption of CML relapse or TKI resistance. In practice, flow cytometry is a key diagnostic tool when a new clonal lymphocytosis is suspected, as demonstrated here.

## Conclusions

We describe a rare case of sequential CLL arising in a patient with CML who was being successfully treated with nilotinib. This case highlights the need for vigilance for secondary hematological malignancies in patients on long-term TKI therapy. The co-occurrence of CML and CLL remains an uncommon clinical scenario, and its pathogenesis is not fully understood. It may represent a chance convergence of two common leukemias, or there may be facilitating factors such as treatment-related immunomodulation or genetic predisposition. In our patient, the emergence of CLL did not necessitate immediate therapy and could be managed conservatively alongside ongoing CML treatment. Prospective monitoring for lymphoproliferative disorders in TKI-treated patients and molecular studies to detect subclinical clones are needed to clarify whether TKI therapy can contribute to secondary lymphoproliferative disorders and to determine the best management practices for patients with dual hematological malignancies.
